# Address by Dr. Edwards

**Published:** 1881-09

**Authors:** 


### ADDRESS BY DR. EDWARDS.

BEFORE THE KENTUCKY STATE DENTAL SOCIETY.

*Mr. Vice-President and Gentlemen.*

The annual revolution of the earth on its axis, the statements of some of our scientists, and the predictions of self constituted prophets as to the dire results to follow the conjunction of several large planets with our earth and the sun, to the contrary notwithstanding, has brought us around to this period of the year 1881 to participate in our eleventh annual meeting, and I congratulate each of my fellow members at the goodly number present with us to-day. It is a subject of congratulation, and a fact to be thankful for, that so far as we are advised our ranks have not been invaded and broken by the omnipresent angel whose dark pinions are ever ready to bear its victim to that other world of which we know so little, and that so many of the *regular*, *true* and *faithful* have met to again clasp the hand of friendship, and to exchange their thoughts and views for the common good of humanity.

The time-honored custom which imposes upon your presiding officer the duty of addressing you at our re-unions is my apology for being before you to-day.

Day after day I have been mindful of this duty imposed by an unwritten law, which there have been innumerable temptations to disregard till I have almost appeared before you to-day without the expected preparation. I trust, however, you will not consider me unappreciative of your confidence and esteem, but will bear with me, whilst for a brief period I invite your attention to some of the questions which I think worthy of your attention, and should secure your hearty co-operation to raise to a higher dignity the moral and intellectual standard for our chosen profession.

Many of you have to-day the honor and pleasure of the acquaintance of a few gentlemen who have seen the rise and progress of Dental Surgery in this country, gentlemen who witnessed the laying of the foundation in the city of Baltimore of the first Dental College in the world. Many, I may say all of you, have seen vast improvements and wonderful discoveries in dentistry. You have seen colleges created, Dental Societies formed and incorporated, and witnessed the birth of journals for the dissemination of knowledge; you are witnessing year by year dental legislation in the several States. Yet, my fellows, Dental Surgery is an infant profession still.

You have a grand work before you—a work demanding your most assiduous attention and study. The field for scientific research in Dental Surgery is vast, and its paths, which are not always smooth or strewn with flowers, are open to all. Let each one of its votaries resolve himself into a committee of one and go forth with his armour on, and battle for the common good of a profession which ministers so largely to the comfort and well-being of our race. There is much truth to be sought for in etiology, pathology, and therapeutics; there are hidden truths to be brought to light, and errors to be revealed by the microscope. A vast field is open before you in the study of preventive dentistry.

I trust many of you will see the day when you will be consulted for advice as to the care of the mouth and teeth, where now you are called on in most instances for specific operations. And if that time comes, what will become of those loathsome parasites who disgrace the profession? What will they know about "professional services rendered"? How will they render their bills? Surely not to sixteen teeth extracted, $2.00 and one set of teeth $6.00!

Their mercantile idea of cost and price will have vanished—Othello's occupation will have gone.

After all that has been written and said on dental education during the past few years, what reforms can you recall? What decided measures have been taken by any of the Dental Colleges looking to a higher degree of proficiency? Are there any? And are the Colleges entirely responsible?

The American system of popular education, as in most other matters is hurried, children are hurried from the nursery to school, hurried through their studies, hurried into business at an immature age, and the idea of push and energy with mind and body on the stretch is exerted to the utmost tension. Consequently the lack of culture, precision, and finish widely prevails.

All professions have suffered much from this hurried system of preparation.

Who are responsible for the young men half educated sent to colleges, in many instances with but little preliminary education and no literary attainments? Is it not frequently the case that physicians and dentists attempt to make professional men of their office boys who may have had the temerity to remark, "I would like to be a doctor," whereupon the employer encourages the idea and possibly renders material assistance? He goes to the professional mill and after two short terms returns with his grist—a full fledged doctor.

How many young men without sufficient intelligence to enter a plain mercantile business are taken into a dental laboratory (not the office) to be made "hewers of wood and drawers of water," to subserve the selfish purposes of the employers (we can not call them preceptors) where they soon acquire the routine work of mounting teeth and moulding the plate, at which they continue until a sufficient number of dollars are accumulated to enable them to go through the form of getting a diploma, or else send their ten dollars on and have one forwarded by return mail, or as is not unfrequently the case fit up a room and dignify it with the name of a Dental Office.

It is not expected nor is it proper that I should go into details or advance arguments in an address of this nature, that is for your consideration and action; though I appeal to every truly professional man and ask if these things are as they should be? Is this the kind of material of which to make professional men? I appeal to you as gentlemen, as lovers and guardians of your profession, to dissuade all young men who may come within the pale of your influence from studying Dental Surgery or any other profession who cannot command the esteem of intelligent and polite people. Elevate the dignity of the profession of your choice and it will command its merited respect.

Though this is the eleventh year of the existence of this Association, the only organization of the kind in the great State of Kentucky, the membership is regretfully small. Each year we have a small accession to our roll, and year by year a few drop out and retire from membership, the one about equaling the other, whereas the former should largely out-number the latter, and the Association should number hundreds where it now numbers tens. Some presumably fall by the wayside for the lack of energy and ambition to keep abreast of the profession, others to compete with their quack neighbors by pretentious advertising at which they are beaten. None can afford to drop out or withhold themselves from membership of some organized dental association; the advantages are apparent to every intelligent and sagacious mind.

I hope some plan may be adopted whereby the better members of the profession throughout the State will be brought into our fold and made co-workers with us in the effort to advance the science and dignity of dentistry, to communicate to each other what we have learned by experience or obtained from other sources of knowledge, which, as true professional men, we would not keep to ourselves, but desire others to share with us for professional advancement and the public good. Gather unto you all who are worthy, but by all means reject those who are unworthy.

I think the profession is in great measure responsible for the positive harm done by this latter class. Unfortunately the people are not sufficiently educated to discriminate between the true professional man and the mere pretender and advertiser. Is it not our duty to enlighten the public mind to enable them to discriminate between the qualified professional man and the charlatan, even though the latter may hold a diploma?

From recent experience in the clerical and executive functions of this Association I have been impressed with the idea that a change in the by-laws by which the officers should hold their office for a longer period than is now provided for, might be advisable. The Secretary for instance, scarcely becomes familiar with his duties before his term of office expires; and so with the President and other officers. For this and for other reasons which many of you doubtless appreciate, I am of the opinion that we might discuss the matter profitably.

Respectfully submitting the foregoing general remarks for your consideration, I will briefly call your attention to some of the more vital subjects for discussion.

In operative dentistry, while "The New Departure" has not made any very great revolution, it has probably brought about a conservatism which may be a factor for good. Amalgams and alloys are coming more boldly to the front, breaking down the barriers of prejudice instilled into the young mind within a few years past; they surely deserve consideration, and in my opinion have their place in dentistry. Tin, owing to its ductility and probable therapeutic effect, used alone or in connection with gold, deserves our consideration.

The comparatively new preparation "Felt Foil" seems to possess qualities admirably adapted to fill certain wants in operative dentistry. The many plastic or cement preparations subserve a most useful purpose in exposed and nearly exposed pulps, but none so far discovered have proven to be more than what we term temporary as filling materials. Gold, the most useful of all the purer metals, still remains the most valuable in dentistry, and as yet stands unrivaled. I do not remember ever to have heard the correction of irregularities mentioned in this Association. Viewing it as of the utmost importance, not only as a matter of æsthetics, but also as a prophylactic, it should be discussed with exhibit of models, etc.

"Tooth grafting" (an improper term, I think, to express the insertion of artificial teeth without plate or pivot in the natural root,) is worthy of experiment.

I would not feel that I had fulfilled my duty as your presiding officer if I failed to call your attention to the existing relationship between the "Goodyear Dental Vulcanite Co." and the Dental Profession. You are aware of the fact that the Cumming's patent expires to-day. Many of you also know of the movement made during the past winter by which Congress was memorialized with the hope of preventing a re-issue of the Cumming's patent. The Company made application for a re-issue. Our counter petitions were presented to Congress, and that body adjourned without taking action in the matter.

Though it is a fact that the Cummings patent is to-day a dead letter, be not deceived as to the facts which exist—the application for re-issue having been made before the patent expired presents it as a living issue for Congress to consider hereafter. I think it would be wise in this and every Association to take some action in the matter with a view to check-mate this rich and long continued monopoly.

I earnestly trust the present session will be harmonious and agreeable as those in the past have been. That those who are with us, though not of us, will be induced to join our ranks and labor in the good cause. That whatever may have been our course in the past we will resolve to do our duty—our whole duty —from this time onward for the common good of humanity and the elevation and dignity of Dental Surgery.

